# Distributed Feedback Laser Based on Tunable Photonic Hypercrystal

**DOI:** 10.3390/ma14154065

**Published:** 2021-07-21

**Authors:** Bartosz Janaszek, Paweł Szczepański

**Affiliations:** 1 Institute of Microelectronics and Optoelectronics, Warsaw University of Technology, Koszykowa 75, 00-665 Warsaw, Poland; pawel.szczepanski@pw.edu.pl; 2 National Institute of Telecommunications, 1 Szachowa Str., 04-894 Warsaw, Poland

**Keywords:** photonic hypercrystals, distributed feedback laser, hyperbolic metamaterials

## Abstract

In this work, we investigate the generation of light in a distributed feedback (DFB) laser composed of periodically arranged layers of hyperbolic medium and active material forming a 1D photonic hypercrystal (PHC). The scope of our study covers the analysis of laser action in the presence of different types of dispersion that are achievable in a hyperbolic medium. Using the example of a PHC structure consisting of graphene-based hyperbolic medium, we demonstrate the possibility of controlling laser action by tuning effective dispersion. Our analysis reveals the possibility of obtaining a single-frequency generation with high side-mode suppression and controllable wavelength of operation. Moreover, we present a new mechanism for the modulation of laser amplitude arising from voltage-controllable dispersion of hyperbolic medium.

## 1. Introduction

The concept of controlling electromagnetic properties by proper design of an artificial structure has been widely investigated over the last few decades. One of the milestones in this field has been achieved by Yablonovitch in his research on photonic crystals (PC) [1,2]. In principle, the control of light propagation in photonic crystals relies on Bragg scattering of light due to a wavelength-scaled periodic spatial variation of the structure resulting in the appearance of a photonic bandgap. To date, extensive scientific efforts dedicated to photonic bandgap materials have proven a wide scope of their applicability, including low-loss resonators, spectral and spatial filters of extreme high quality-factor, highly efficient light emitters, chemical and biological sensors, tunable lasers, reflective full-color displays, components for signal processing, and many other practical implementations [3,4,5,6,7]. On the other hand, the metamaterial platform that relies on the average response originating from structurization of dimensions much smaller than the freespace wavelength of light has been considered as another promising way to obtain control over the light propagation [8,9]. To date, these two approaches, which were previously considered to be mutually exclusive due to geometrical-scale separation, have been successfully employed to form basic building blocks of novel passive and active photonic devices [10].

More recently, a new class of artificial optical media, i.e., photonic hypercrystals, combining advantageous photonics crystals and optical metamaterial, has been proposed by Narimanov in his work [10]. To overcome the problem of scale separation, an application of a special class of optical metamaterials, so-called hyperbolic metamaterials (HMM), has been proposed. This class of structure, typically realized in the simple multilayer form, reveals unique dispersion properties that allow the obtaining of many practical functionalities, such as tunable spectral filters [11], perfect absorbers [12], and many others [13,14,15,16,17,18]. Since the pioneering work of Narimanov published in 2014 [10], the topic of photonic hypercrystals (PHCs) has been investigated both theoretically [19,20,21,22,23,24,25,26,27,28,29,30] and experimentally [31]. It has been demonstrated that surface waves in a hypercrystal combine properties of Tamm states in the photonic crystal and surface plasmon polaritons at the metal–dielectric interface [10,30]. Many scientific efforts have been also focused on applying PHC structures in difractionless imaging [19], efficient polarizers [32], and cloaking applications [20]. It has been also shown that a photonic hypercrystal slab may act as a perfect absorber [21]. Moreover, studies in this field also uncovered that PHC structures reveal a remarkable potential for spontaneous emission enhancement combined with effective light outcoupling compared to hyperbolic metamaterials [33,34]. Unusual propagation properties of surface waves in hypercrystals have attracted widespread scientific attention [22,23,24,26,27,28]. To date, propagation of surface waves in hypercrystals has been considered in many different schemes, including propagation in the presence of an external magnetic field [22], various interfaces [26], and aperiodical PHC geometries [24] as well as when employing plasmon polariton gap to obtain optical bistability [27] or possibility to excite electrostatic waves [28]. In particular, it has been shown that the propagation of surface waves may be controlled in photonic hypercrystals based on graphene [23]. An important contribution in the field of hypercrystals was published by Smolyaninov in 2019 [25]. In his work, Smolyaninov demonstrated that the level of nonlinearities achievable in photonic hypercrystals paves promising foundations for future applications in optical limiting and optical computing [25]. To date, the majority of presented works have been focused on slab (one-dimensional) hypercrystals [19,20,21,22,23,24,26,27], which are regarded as highly feasible. It has been reported that HPCs are able to operate over visible and infrared frequencies [35]. Recently, it has been also experimentally demonstrated that, apart from conventional deposition techniques, optical binding may be employed in controllable ways to organize hyperbolic metamaterial slabs into photonic hypercrystal [29].

In this study, for the first time, we investigated the lasing phenomenon in a DFB laser based on photonic hypercrystals. In our analysis, isotropic medium with optical gain and tunable hyperbolic metamaterial (HMM) together form the periodical arrangement required to obtain the distributed feedback loop. The tunability of the HMM structure is provided by the incorporation of graphene, which is sensitive to variations in temperature and/or an external static magnetic/electric field [36]. We investigated the possibility of controlling a threshold modal spectrum enabled with tunable dispersion of the HMM structure. For this purpose, we developed an original approach for threshold lasing analysis based on the transfer matrix method. We demonstrated that switching the dispersion type of HMM medium may lead to a number of interesting effects, such as single-mode lasing with a voltage-controlled frequency of generation. Moreover, using the example of a sample PHC laser, we presented a new mechanism for modulation of laser amplitude arising from the dispersion properties of a hyperbolic medium.

## 2. Theory

Here, we explicitly present our approach for the analysis of threshold generation in a DFB laser based on photonic hypercrystals. The scope of this section covers a description of the numerical model of the PHC structure and propagation in such media, as well as a definition of the assumed threshold lasing condition.

### 2.1. DFB Laser Based on Photonic Hypercrystal

**Figure 1:**
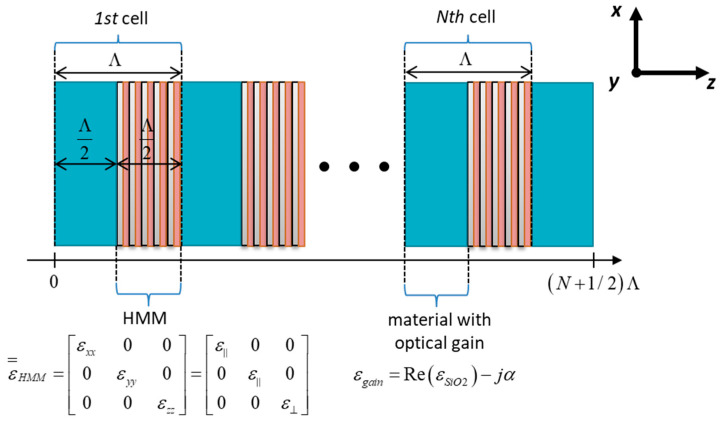
Schematic representation of the considered PHC structure.

Typically, a photonic hypercrystal is realized as a periodical arrangement of hyperbolic metamaterial and a dielectric medium. For the purpose of lasing threshold analysis, we assume that our PHC is composed of alternating layers of hyperbolic medium and material with optical gain (see Figure 1). Additionally, to obtain symmetrical boundary conditions for output radiation, the considered PHC structure is truncated on both sides with the gain material.

**Figure 2:**
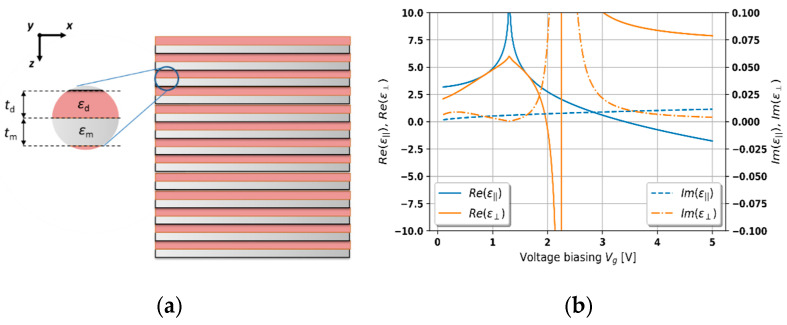
Schematic of the considered HMM medium (**a**) and effective permittivity components of the considered HMM medium vs. voltage biasing for wavelength *λ*_0_ = 1550 nm (**b**).

The gain material is described with εgain=Re(εSiO2)−jα, where $\varepsilon_{SiO2}$ corresponds to the permittivity of fused silica [37] and α is a parameter corresponding to net gain of the material. In our analysis, the parameters of the gain material correspond to parameters of erbium-doped fused silica thin film [38]. Moreover, the multilayer HMM medium is considered as a homogenous uniaxial anisotropic medium with effective permittivity tensor components acquired with the help of well-established effective medium theory [12,39]:

(1) $$\varepsilon_{∥}=\varepsilon_{xx}=\varepsilon_{yy}=\frac{t_{d}\varepsilon_{d}+t_{m}\varepsilon_{m}}{t_{d}+t_{m}},$$

(2) $$\varepsilon_{⊥}=\varepsilon_{zz}=\frac{\varepsilon_{d}\varepsilon_{m}\left(t_{d}+t_{m}\right)}{t_{d}\varepsilon_{m}+t_{m}\varepsilon_{d}},$$

where $\varepsilon_{m}/\varepsilon_{d}$ and $t_{m}/t_{d}$ are related to the permittivity and thickness of metal/dielectric layers constituting the unit cell of the HMM structure (see Figure 2a). In our analysis, we assume that the HMM structure is a periodical arrangement of a six-monolayer graphene sheet with complex permittivity described by the Kubo formula [36] (i.e., $\varepsilon_{m}=\varepsilon_{graphene}(\lambda )$ and $t_{m}$ = 6 × 0.35 nm = 2.1 nm [40]) and 4-nm-thick hafnium oxide (HfO_2_) layer (i.e., $\varepsilon_{d}=\varepsilon_{HfO2}(\lambda )$ and $t_{d}$ = 4 nm) with the permittivity expressed by the Sellmeier equation [41]. It is worth highlighting that the assumed dimension of the unit cell, i.e., $t=t_{d}+t_{m}$, of the HMM structure is much smaller than the wavelength considered, which validates the effective-medium approach as a method for the description of effective parameters of the structure [12,34].

It has been demonstrated that graphene is sensitive to an external electric field [36]. Due to that property, it is possible to tune the effective permittivity of a hyperbolic medium based on graphene [11,40]. The influence of voltage biasing on the effective permittivity components of the considered HMM structure is illustrated in Figure 2b. It can be observed that for a chosen wavelength *λ*_0_ = 1550 nm, it is possible to obtain various types of dispersion, namely, elliptic ($\varepsilon_{∥}>0$ and $\varepsilon_{⊥}>0$), epsilon-near-zero(ENZ) ($0<\varepsilon_{∥}<1$ or $0<\varepsilon_{⊥}<1$), and types I ($\varepsilon_{∥}>0$ and $\varepsilon_{⊥}<0$) and II hyperbolic ($\varepsilon_{∥}<0$ and $\varepsilon_{⊥}>0$) dispersion, depending on the voltage applied (see Figure 2b). It is noteworthy that all employed materials were chosen as examples to demonstrate more general phenomena arising in a PHC-based laser. However, the feasibility of the proposed structure was considered in the analysis. In particular, similar graphene-based multilayer stacks have been experimentally demonstrated [31,42]. Additionally, the deposition process of active material deposition, e.g., Er-doped glass, may be realized by means of a compatible technology, e.g., RF magnetron sputtering [38,43].

### 2.2. Transfer Matrix Method

Propagation in the considered system can be described with a transfer matrix method suitable for anisotropic media. Assuming the normalized magnetic field $\vec{\bar{H}}=-j\sqrt{\frac{\mu_{0}}{\varepsilon_{0}}}\vec{H}$, Maxwell equations may be written in the following form:

(3) $$\begin{array}{cc} \nabla \times \vec{E}=k_{0}\vec{\bar{H}} & \nabla \times \vec{\bar{H}} \end{array}=k_{0}\vec{E}$$

Using Equation (2) for fields $\begin{array}{cc} \vec{E}(\vec{r})={\vec{E}}_{0}e^{jk_{x}x}e^{jk_{y}y}e^{jk_{z}z} & \vec{\bar{H}}(\vec{r})={\vec{\bar{H}}}_{0}e^{jk_{x}x}e^{jk_{y}y}e^{jk_{z}z} \end{array}$, where $e^{+jkz}$ denotes +*z* direction propagation and $k_{x,y,z}$ are components of the wavevector, the problem of wave propagation in a nonmagnetic biaxial anisotropic medium may be formulated in the form of the matrix equation [44]:

(4) $$\frac{\partial \psi }{\partial z^{\prime }}-\Omega \psi =0$$

where

(5) $$\Omega =\left[\begin{array}{cccc} 0 & 0 & \frac{1}{\varepsilon_{z}}\bar{k_{x}}\bar{k_{y}} & \mu_{y}-\frac{{\bar{k_{x}}}^{2}}{\varepsilon_{z}}\\ 0 & 0 & \frac{{\bar{k_{y}}}^{2}}{\varepsilon_{z}}-\mu_{x} & -\frac{1}{\varepsilon_{z}}\bar{k_{x}}\bar{k_{y}}\\ \frac{1}{\mu_{z}}\bar{k_{x}}\bar{k_{y}} & \varepsilon_{y}-\frac{{\bar{k_{x}}}^{2}}{\mu_{z}} & 0 & 0\\ \frac{{\bar{k_{y}}}^{2}}{\mu_{z}}-\varepsilon_{x} & -\frac{1}{\mu_{z}}\bar{k_{x}}\bar{k_{y}} & 0 & 0 \end{array}\right],$$

and

(6) $$\psi =\left[\begin{array}{c} E_{x}(z^{\prime })\\ E_{y}(z^{\prime })\\ \bar{H_{x}}(z^{\prime })\\ \bar{H_{y}}(z^{\prime }) \end{array}\right],$$

and $z^{\prime }=z/k_{0}$, $\bar{k_{i}}=k_{i}/k_{0}$ for i=x,y. It is worth noting that in this approach, absorption/gain of material is described with an imaginary part of permittivity. The solution of Equation (4) can be expected to take the form of an equation with matrix function, which can be reformulated as

(7) $$\psi (z^{\prime })=We^{\lambda z^{\prime }}c$$

where $c=W^{-1}\psi (0)$ is a column vector containing the amplitude coefficients normalized to unity for each mode, while W and λ are eigenvector and eigenvalue matrices of the characteristic matrix Ω.

**Figure 3:**
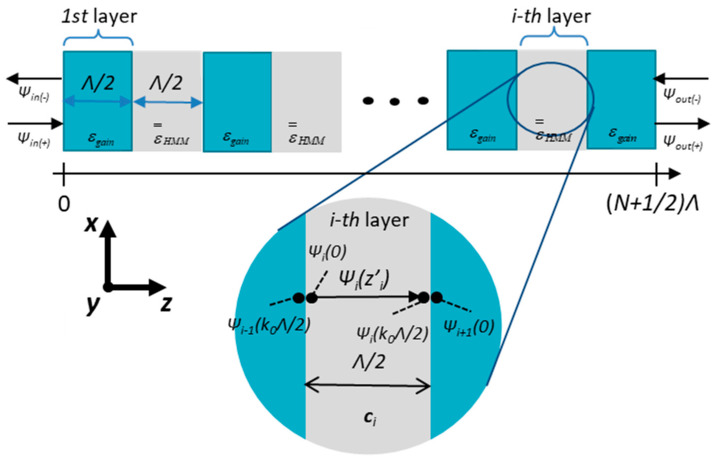
Schematic of the considered PHC structure in transfer matrix method analysis.

By imposing boundary conditions at the interface of *i*-th layer (see Figure 3), we obtain

(8) $$\psi_{i-1}(k_{0}L_{i-1})=\psi_{i}(0),$$

(9) $$\psi_{i}(k_{0}L_{i})=\psi_{i+1}(0),$$

(10) $$W_{i-1}e^{{}_{\lambda_{i-1}k_{0}L_{i-1}}}c_{i-1}=W_{i}c_{i},$$

(11) $$W_{i}e^{{}_{\lambda_{i}k_{0}L_{i}}}c_{i}=W_{i+1}c_{i+1},$$

which allows us to formulate the relationship between amplitudes entering and exiting the *i*-th layer:

(12) $$c_{i+1}=T_{i}\cdot c_{i},$$

(13) $$T_{i}=W_{i+1}^{-1}W_{i}e^{\lambda_{i}k_{0}L_{i}},$$

where $T_{i}$ is the transfer matrix of the *i*-th layer. By considering the influence of the surrounding medium, as well as multiplying matrices of subsequent layers (starting from the last layer), the matrix describing the complete structure may be obtained:

(14) $$T_{global}=W_{N+1}^{-1}W_{N}e^{\lambda_{N}k_{0}L_{N}}\cdot W_{N}^{-1}W_{N-1}e^{\lambda_{N-1}k_{0}L_{N-1}}\cdot \ldots \cdot W_{2}^{-1}W_{1}e^{\lambda_{1}k_{0}L_{1}}\cdot W_{1}^{-1}W_{0},$$

where $W_{0}$ and $W_{N+1}$ are eigenvector matrices of media surrounding the structure. Knowing the form of the global matrix, we may formulate an expression binding the amplitude coefficients at both sides of the structure:

(15) $$c_{out}=T_{global}\cdot c_{in}$$

where $c_{in}$ and $c_{out}$ are amplitude coefficients of modes entering and exiting the structure, respectively (see Figure 3).

### 2.3. Analysis of Threshold Generation in DFB Laser

**Figure 4:**
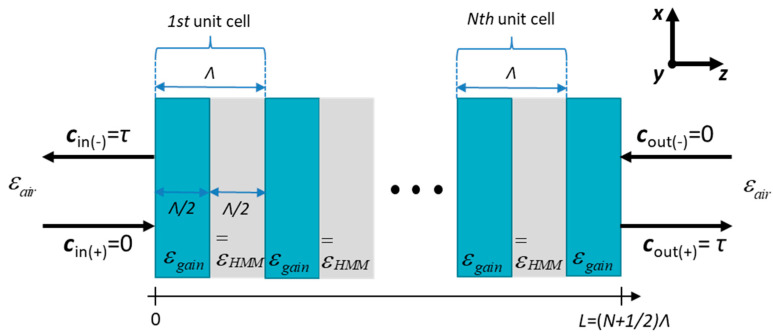
The PHC laser operating at threshold.

To analyze threshold generation, we consider a DFB laser based on a PHC structure truncated with gain medium on both sides (see Figure 4), providing identical nonzero internal reflections, i.e., $\rho_{\mathrm{int}}$ ≈ 0.18, and forming a Fabry–Perot cavity. Thus, the proposed structure may be considered as a DFB laser with end reflectors. The unit cell of the laser structure consists of isotropic gain material, i.e., $\varepsilon_{x}=\varepsilon_{y}=\varepsilon_{z}=\varepsilon_{gain}$, and uniaxially anisotropic HMM medium, i.e., $\varepsilon_{x}=\varepsilon_{y}=\varepsilon_{∥}$ and $\varepsilon_{z}=\varepsilon_{⊥}$; see Equations (1) and (2). The periodical arrangement of the layers constituting the PHC structure provides the main mechanism for the feedback loop, i.e., distributed feedback. It is worth noting that we limit our analysis to propagation along the *z* axis, i.e., $k_{x}=k_{y}=0$

In our analysis, we consider laser action to be the result of a self-oscillating character of the proposed structure. Thus, there are no incoming waves from outside of the structure, while the internal waves start with nonzero amplitude arising from a reflection at the gain medium/air interface and further accumulating energy via scattering from counter-propagating waves inside the structure [45]. This state may be formulated in the form of boundary conditions, identical for both possible polarizations, which is consistent with the employed TMM approach:

(16) $$c_{in(-)(TE/TM)}=c_{in(-)}=c_{out(+)(TE/TM)}=c_{out(+)}=\tau$$

(17) $$c_{in(+)(TE/TM)}=c_{in(+)}=c_{out(-)(TE/TM)}=c_{out(-)}=0$$

where τ is the Fresnel transmission amplitude coefficient for the interface between the considered gain medium and air. Finally, knowing the global matrix of the structure (see Equation (9)), it is possible to formulate the generation condition as follows:

(18) $$\left[\begin{array}{c} \tau \\ 0\\ \tau \\ 0 \end{array}\right]=T_{global}\cdot \left[\begin{array}{c} 0\\ \tau \\ 0\\ \tau \end{array}\right],$$

Additionally, for the purpose of the modal analysis, we defined the Bragg wavelength of the PHC structure as follows:

(19) $$\lambda_{bragg}=2\cdot \Lambda \cdot \left(\left|n_{HMM}\right|+\left|n_{g}\right|\right),$$

where $n_{HMM}$ and $n_{g}$ are refractive indices of the HMM medium and material with optical gain, respectively, while Λ denotes the thickness of the unit cell of the PHC structure (see Figure 4). Finally, the amplitude gain coefficient is calculated based on the imaginary part of refractive index of the gain medium required to satisfy the threshold condition for a given mode:

(20) g=2Im(ng)λ0,

where $\lambda_{0}$ is the freespace wavelength of the mode.

## 3. Results and Discussion

The analysis in the first subsection is focused on the influence of various dispersion types of hyperbolic medium on the output lasing characteristics of the complete PHC-based laser. The second section is dedicated to the possibility of controlling lasing action in a DFB laser by tuning the dispersion properties of a hyperbolic medium.

### 3.1. Threshold Characteristics of DFB Laser Based on PHC Structure

Here, we investigate tunable lasing in a PHC structure. The tunability of the laser is provided by incorporating a hyperbolic medium sensitive to external stimulus; in this case, we employ a voltage-sensitive, graphene-based HMM structure. Within our analysis, we demonstrate threshold generation characteristics for various types of dispersion of the HMM structure. For each type of dispersion, i.e., elliptic, type I hyperbolic, epsilon-near-zero (ENZ), and type II hyperbolic dispersion, we demonstrate two different characteristics calculated for various electric biasing. In our analysis, we assume that effective permittivity components are constant within the considered spectral range. Additionally, we used normalized spectroscopic units, i.e., wavelengths normalized to the Bragg wavelength $\lambda_{bragg}$ = 1.55 μm, which imply a different thickness of the unit cell Λ for each voltage biasing; see Equation (19). It is worth noting that voltage-dependent optical losses of graphene, i.e., the imaginary part of permittivity, and, consequently, effective absorption of the hyperbolic medium (see the imaginary part of effective permittivity components in Figure 2b), were considered in the analysis. Since only the propagation along *z* axis is considered, all possible polarizations of light perceive the same optical properties, i.e., permittivity, of the hyperbolic medium. Thus, all obtained threshold properties are polarization invariant. Since two different feedback loop mechanisms exist in the considered laser, i.e., the Fabry–Perot cavity and distributed feedback (see Figure 4), it can be expected that the observed effects will be an aggregated outcome of those two mechanisms. Moreover, distributed feedback, which is a result of coupling between counter-propagating waves, may be achieved via index (periodical variation of refractive index along the structure) or gain coupling (periodical variation of gain along the structure). In our analysis, the laser structure reveals both coupling mechanisms simultaneously, which, depending on the biasing voltage, are more or less dominant at a time.

**Figure 5:**
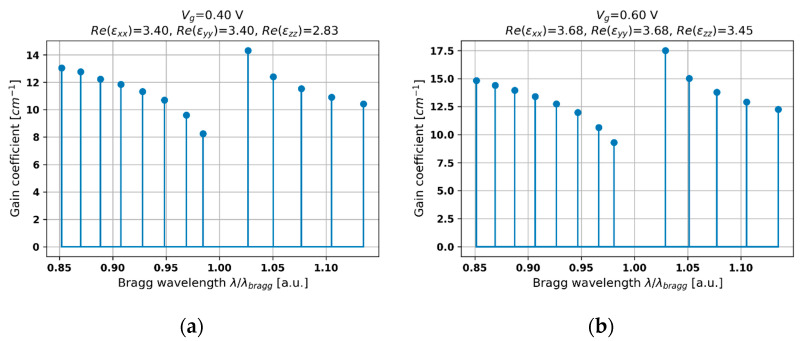
Threshold modal spectra for PHC structures with HMM structures revealing elliptic dispersion and biased with external voltage *V_g_* = 0.4 V (**a**) and 0.6 V (**b**).

Let us start the analysis from the case of the hyperbolic medium revealing elliptic dispersion, which is achievable for, e.g., $V_{g}$ = 0.4 V and 0.6 V (see Figure 5a,b). Since the contrast between the refractive indices of media constituting the unit cell of the structure is substantial, the obtained characteristics reveal properties similar to an index-coupled DFB laser. In particular, a frequency bandgap, which is a distinctive feature of an index-modulated DFB laser, can be observed (see Figure 5a). However, by increasing voltage biasing, we obtain a higher index contrast and higher level of lasing threshold, which is not in accordance with the behavior of a DFB laser [45]. Typically, increasing the refractive index (permittivity) contrast between layers constituting the unit cell of a DFB laser results in a stronger index modulation and lower level of the lasing threshold. Nonetheless, in our case, by increasing the voltage up to 0.6 V, we also obtain higher absorption (see Figure 2b), which causes an overall increase in the lasing threshold level (see comparison in Figure 5a,b. Thus, under the considered conditions, the similarity between the obtained characteristics and the behavior of a refractive-index-modulated DFB laser [46] confirms the validity of the proposed approach. In this case, the influence of the Fabry–Perot cavity may be considered to be negligible. 

**Figure 6:**
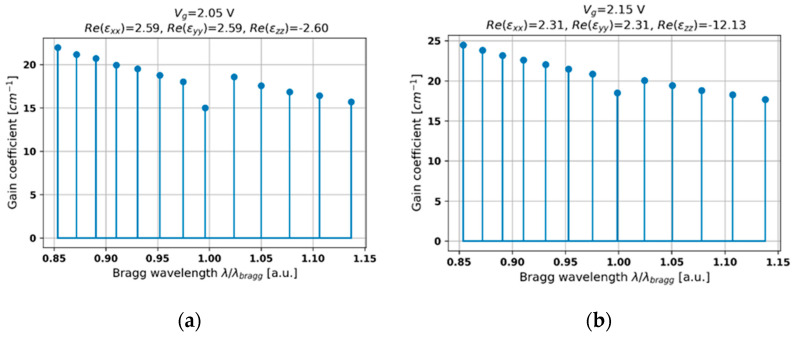
Threshold modal spectra for PHC structures with HMM structures revealing type I hyperbolic dispersion and biased with external voltage *V_g_* = 2.05 V (**a**) and 2.15 V (**b**).

Further increase in the voltage biasing leads to type I hyperbolic dispersion of the HMM structure (see Figure 2b). In this case, the obtained characteristics reveal a regular distribution of modes, which may be related to the low index contrast and dominant influence of the Fabry–Perot cavity. Additionally, the obtained characteristics reveal a central mode with the lowest threshold located near the Bragg wavelength arising from the fact that gain coupling plays a more significant role in shaping the overall generation properties (see Figure 6a,b). It can be observed that a higher level of gain is required to initiate lasing (compare Figure 5 and Figure 6), which is a result of increased effective absorption (see Figure 2b). Moreover, by tuning biasing voltage, it is possible to adjust the level of the lasing threshold as well as shifting the spectral position of the central mode (compare Figure 6a,b). Hence, by adjusting the biasing voltage and the gain coefficient of the active medium, it is feasible to obtain a single-frequency generation.

**Figure 7:**
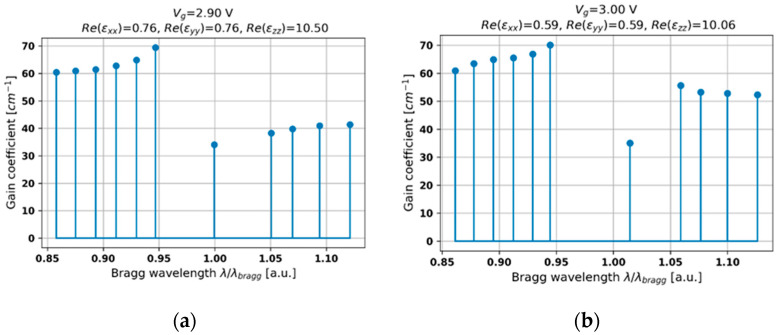
Threshold modal spectra for PHC structures with HMM structures revealing epsilon-near-zero dispersion and biased with external voltage *V_g_* = 2.9 V (**a**) and 3.0 V (**b**).

Inducing the ENZ dispersion in the hyperbolic medium leads to a further increase in the gain level that is required to obtain generation (see Figure 7a,b). Such a high lasing threshold is the combined effect of a high level of effective optical losses for the given biasing voltage (see Figure 2b) and a relatively weak gain/loss coupling [45]. Despite the weak gain coupling, this mechanism has the biggest influence on the output characteristics, which can be observed in the occurrence of the central mode. Moreover, modes of wavelength that are shorter than the Bragg wavelength reveal a higher lasing threshold, which is an opposite tendency with respect to the elliptic dispersion case (compare Figure 5a and Figure 7a). This effect is related to the inverse relation between the value of refractive indices of layers constituting the PHC structure. Additionally, we can observe a central mode with a voltage-controllable spectral position and relatively high side-mode suppression ratio (see Figure 7a). Thus, again, a stable, single-mode operation of controllable wavelength generation may be obtained.

**Figure 8:**
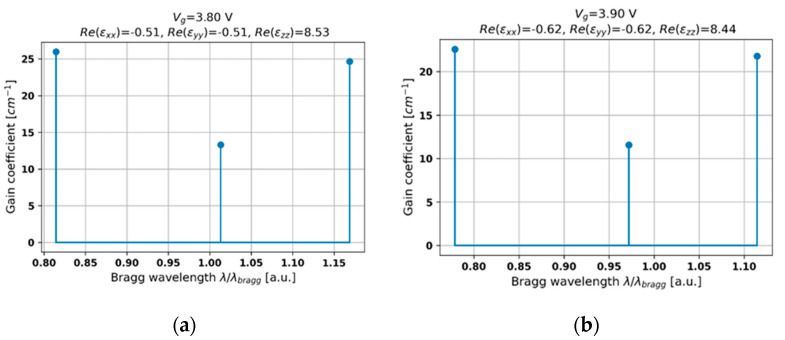
Threshold modal spectra for PHC structures with HMM structures revealing type II hyperbolic dispersion and biased with external voltage *V_g_* = 3.8 V (**a**) and 3.9 V (**b**).

Finally, let us consider the influence of type II hyperbolic dispersion on threshold generation in a PHC laser (see Figure 8a,b). Again, in this case, the distributed feedback arises from a strong gain/loss modulation, while the influence of the Fabry–Perot cavity is negligible. Moreover, since type II hyperbolic dispersion reveals a low optical density and a high gain/loss modulation, the observed modes are sparsely distributed. Furthermore, the difference in the level of thresholds between the central and adjacent modes is substantial. Thus, the PHC laser operating under such conditions reveals a high spectral and power side-mode suppression ratio, which is very promising in terms of obtaining a stable and low-loss single-frequency operation. Additionally, by increasing biasing voltage, it is possible to shift the spectral position of the central mode and, due to a lower effective absorption (see Figure 2b), to lower the lasing threshold (compare Figure 8a,b).

### 3.2. Controlling Generation in a Single Tunable PHC Structure

Now, we demonstrate the possibility of controlling the generation properties of a PHC laser by demonstrating a sample scenario, i.e., a low-threshold, single-frequency generation with a voltage-controllable wavelength. As an example, we chose a PHC structure with a unit cell composed of two Λ = 945 nm layers consisting of a hyperbolic medium and active material with optical gain (see Figure 3). The active medium in this example has a homogenously broadened gain curve with a resonance wavelength $\lambda_{lorentz}$= 1560 nm, fullwidth at half maximum $\lambda_{FWHM}$ = 6.2 nm, and maximum $g_{0}$ = 500 cm^−1^, which correspond to the emission line of erbium-doped SiO_2_ glass [47].

**Figure 9:**
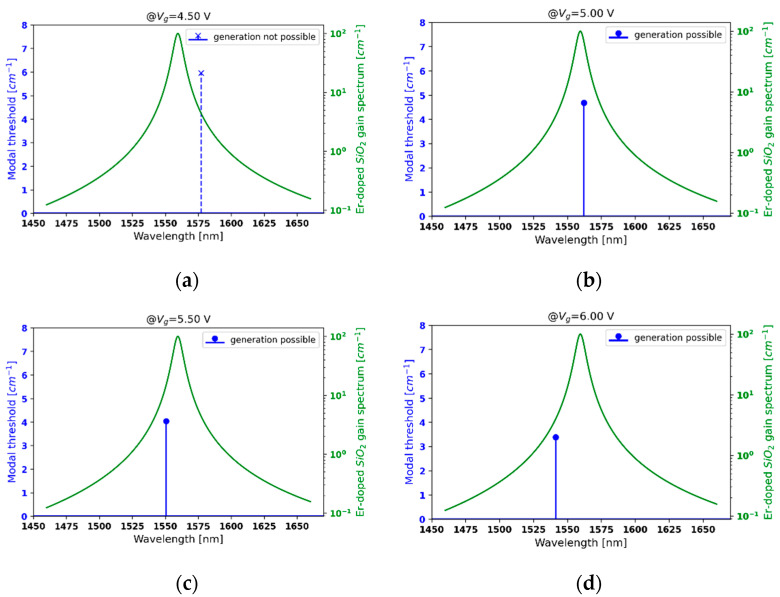
Threshold modal spectra for a PHC structures with HMM structures revealing type II hyperbolic dispersion and biased with external voltage *V_g_* = 4.5 V (**a**), 5.0 V (**b**), 5.5 V (**c**) and 6.0 V (**d**).

For the purpose of this analysis, the gain curve of the active medium was plotted together with the modal spectrum of the considered PHC laser for different voltage biasing providing the type II hyperbolic dispersion (see Figure 9a–d). As can be observed, the sparse modal spectrum arising from the strong gain/loss modulation provided by type II hyperbolic dispersion leads to single-mode operation. It is worth noting that for the given gain curve, the generation condition, i.e., the modal lasing threshold is higher than gain provided by the active medium, is not satisfied for each voltage biasing (see Figure 9a), which is caused by the intrinsic absorption of graphene. However, increasing biasing voltage leads to a lower absorption and higher modulation depth (see Figure 2b) and, thus, a lower level of the lasing threshold. Moreover, by changing the voltage from 4.5 V to 6 V, it is possible to influence the interplay between the gain/loss and index modulations of the DFB laser, which affects the spectral position of the central mode (compare Figure 9a–d). Thus, by adjusting the voltage biasing, it is possible to select the generated frequency or to break the lasing (see Figure 9a–d). It is noteworthy that the possibility of switching off the laser action by changing the voltage biasing delivers an intrinsic mechanism for voltage-controlled amplitude modulation. Thus, the proposed PHC structure provides a means for achieving single-mode operation with a controllable generation frequency, which can be utilized in optical metrology.

## 4. Conclusions

For the first time, we investigated lasing in a novel class of hybrid metamaterials based on hyperbolic media, namely, photonic hypercrystals, which, according to our knowledge, has not been yet investigated. To calculate the threshold characteristics of PHC laser, we derived and explicitly demonstrated a TMM-based approach. As an example, we utilized a hyperbolic structure based on graphene, providing voltage tunability of the complete PHC laser. The scope of our analysis covered generation properties arising from the various dispersion types of the hyperbolic medium and the possibility of controlling them with the help of an external stimulus, i.e., an external electric field. We demonstrated that it is possible to obtain a single-mode generation with a voltage-controllable wavelength of operation, which may be useful in many applications related to optical metrology. Additionally, we demonstrated that a considered PHC laser reveals the intrinsic internal amplitude modulation mechanism. It is worth noting that all the presented features are not the consequence of any unique material properties and may be replicated with the use of media revealing similar optical properties.

## Data Availability

All reported data and tools are available on request.

## References

[1] Yablonovitch E., Leung K.M. (1991). Hope for Photonic Bandgaps. Nature.

[2] Yablonovitch E. (1994). Photonic Crystals. J. Mod. Opt..

[3] Soukoulis C.M. (1996). Photonic Band Gap Materials.

[4] Passaro V. (2013). Advances in Photonic Crystals.

[5] Blanco Á., López C. (2006). Photonic Crystals: Fundamentals and Applications. Annual Review of Nano Research.

[6] Wang M., Zou C., Sun J., Zhang L., Wang L., Xiao J., Li F., Song P., Yang H. (2017). Asymmetric Tunable Photonic Bandgaps in Self-Organized 3D Nanostructure of Polymer-Stabilized Blue Phase I Modulated by Voltage Polarity. Adv. Funct. Mater..

[7] Lu Y., Yang Y., Wang Y., Wang L., Ma J., Zhang L., Sun W., Liu Y. (2018). Tunable liquid-crystal microshell-laser based on whispering-gallery modes and photonic band-gap mode lasing. Opt. Express.

[8] Hess O., Pendry J.B., Maier S.A., Oulton R.F., Hamm J.M., Tsakmakidis K.L. (2012). Active Nanoplasmonic Metamaterials. Nat. Mater..

[9] Smith D.R., Pendry J.B. (2006). Homogenization of Metamaterials by Field Averaging (Invited Paper). J. Opt. Soc. Am. B.

[10] Narimanov E.E. (2014). Photonic Hypercrystals. Phys. Rev. X.

[11] Kieliszczyk M., Janaszek B., Tyszka-Zawadzka A., Szczepański P. (2018). Tunable Spectral and Spatial Filters for the Mid-Infrared Based on Hyperbolic Metamaterials. Appl. Opt..

[12] Xiang Y., Dai X., Guo J., Zhang H., Wen S., Tang D. (2015). Critical Coupling with Graphene-Based Hyperbolic Metamaterials. Sci. Rep..

[13] Baqir M.A., Farmani A., Fatima T., Raza M.R., Shaukat S.F., Mir A. (2018). Nanoscale, Tunable, and Highly Sensitive Biosensor Utilizing Hyperbolic Metamaterials in the near-Infrared Range. Appl. Opt..

[14] Sreekanth K.V., Alapan Y., ElKabbash M., Ilker E., Hinczewski M., Gurkan U.A., De Luca A. (2016). Extreme sensitivity biosensing platform based on hyperbolic metamaterials. Nat. Mater..

[15] Autore M., Li P., Dolado I., Alfaro-Mozaz F.J., Esteban R., Atxabal A., Casanova F., Hueso L.E., Alonso-Gonzalez P., Aizpurua J. (2018). Boron nitride nanoresonators for phonon-enhanced molecular vibrational spectroscopy at the strong coupling limit. Light Sci. Appl..

[16] Shkondin E., Repan T., Aryaee Panah M.E., Lavrinenko A.V., Takayama O. (2018). High Aspect Ratio Plasmonic Nanotrench Structures with Large Active Surface Area for Label-Free Mid-Infrared Molecular Absorption Sensing. ACS Appl. Nano Mater..

[17] Janaszek B., Kieliszczyk M., Tyszka-Zawadzka A., Szczepański P. (2018). Multiresonance Response in Hyperbolic Metamaterials. Appl. Opt..

[18] Guo Y., Newman W., Cortes C.L., Jacob Z. (2012). Applications of Hyperbolic Metamaterial Substrates. Adv. OptoElectronics.

[19] Huang Z., Narimanov E.E. (2014). Optical Imaging with Photonic Hyper-Crystals: Veselago Lens and Beyond. J. Opt..

[20] Wang J.-R., Chen X.-D., Zhao F.-L., Dong J.-W. (2016). Full Polarization Conical Dispersion and Zero-Refractive-Index in Two-Dimensional Photonic Hypercrystals. Sci. Rep..

[21] Chang Y.-C., Kildishev A.V., Narimanov E.E., Norris T.B. (2016). Metasurface Perfect Absorber Based on Guided Resonance of a Photonic Hypercrystal. Phys. Rev. B.

[22] Fedorin I.V. (2018). Electrodynamic Properties of a Hypercrystal with Ferrite and Semiconductor Layers in an External Magnetic Field. Superlattices Microstruct..

[23] Cheng M., Fu P., Lin Y., Chen X., Chen S., Tang X., Feng S. (2018). Analysis of Tunable and Highly Confined Surface Wave in the Photonic Hypercrystals Containing Graphene-Based Hyperbolic Metamaterial. Superlattices Microstruct..

[24] Paim M.C., Isidio de Lima J.J., Rodriguez-Esquerre V.F. Propagation Properties of Fibonacci Hypercrystal Based on Metamaterials. Proceedings of the Metamaterials, Metadevices, and Metasystems 2018.

[25] Smolyaninov I.I. (2019). Nonlinear Optics of Photonic Hyper-Crystals: Optical Limiting and Hyper-Computing. J. Opt. Soc. Am. B.

[26] Fedorin I. (2019). Surface Electromagnetic Waves at the Interface between Dissipative Porous Nanocomposite and Hypercrystal under Different Temperatures. Phys. Lett. A.

[27] Ali M.Z. (2021). Plasmon-Polariton Gap and Associated Phenomenon of Optical Bistability in Photonic Hypercrystals. Phys. Lett. A.

[28] Moradi A. (2021). Electrostatic Waves in Photonic Hypercrystals. Phys. Lett. A.

[29] Cui L., Du G., Ng J. (2020). Angle-Independent and -Dependent Optical Binding of a One-Dimensional Photonic Hypercrystal. Phys. Rev. A.

[30] Wu F., Lyu K., Hu S., Yao M., Xiao S. (2021). Ultra-Large Omnidirectional Photonic Band Gaps in One-Dimensional Ternary Photonic Crystals Composed of Plasma, Dielectric and Hyperbolic Metamaterial. Opt. Mater..

[31] Brouillet J., Papadakis G.T., Atwater H.A. (2019). Experimental Demonstration of Tunable Graphene-Polaritonic Hyperbolic Metamaterial. Opt. Express.

[32] Wu F., Xiao S., Liu D., Chen Z., Chen G., Peng X. (2021). Complete Redshift Photonic Bandgap and Dual-Wavelength Polarization Selection in Periodic Multilayer Structure Containing Hyperbolic Metamaterial. Opt. Commun..

[33] Galfsky T., Sun Z., Considine C.R., Chou C.-T., Ko W.-C., Lee Y.-H., Narimanov E.E., Menon V.M. (2016). Broadband Enhancement of Spontaneous Emission in Two-Dimensional Semiconductors Using Photonic Hypercrystals. Nano Lett..

[34] Galfsky T., Gu J., Narimanov E.E., Menon V.M. (2017). Photonic Hypercrystals for Control of Light–Matter Interactions. Proc. Natl. Acad. Sci. USA.

[35] Carvalho M.C., Isidio de Lima J.J., Rodriguez-Esquerre V.F. Multilayered Metamaterials Hypercrystals at Visible and Infrared Frequencies. Proceedings of the Metamaterials, Metadevices, and Metasystems 2019.

[36] Hanson G.W. (2008). Dyadic Green’s Functions for an Anisotropic, Non-Local Model of Biased Graphene. IEEE Trans. Antennas Propag..

[37] Malitson I.H. (1965). Interspecimen Comparison of the Refractive Index of Fused Silica. J. Opt. Soc. Am..

[38] Cattaruzza E., Battaglin G., Muzio M., Riello P., Trave E. (2010). Er-Doped Dielectric Films by Radiofrequency Magnetron Co-Sputtering. Surf. Coat. Technol..

[39] Choy T.C. (2016). Effective Medium Theory: Principles and Applications (International Series of Monographs on Physics).

[40] Janaszek B., Tyszka-Zawadzka A., Szczepański P. (2016). Tunable Graphene-Based Hyperbolic Metamaterial Operating in SCLU Telecom Bands. Opt. Express.

[41] Al-Kuhaili M.F. (2004). Optical Properties of Hafnium Oxide Thin Films and Their Application in Energy-Efficient Windows. Opt. Mater..

[42] Wu F., Lu G., Xue C., Jiang H., Guo Z., Zheng M., Chen C., Du G., Chen H. (2018). Experimental Demonstration of Angle-Independent Gaps in One-Dimensional Photonic Crystals Containing Layered Hyperbolic Metamaterials and Dielectrics at Visible Wavelengths. Appl. Phys. Lett..

[43] Cattaruzza E., Battaglin G., Visentin F., Trave E. (2009). Er-Doped SiO2 Films by Rf Magnetron Co-Sputtering. J. Non Cryst. Solids.

[44] Janaszek B., Kieliszczyk M., Tyszka-Zawadzka A., Szczepański P. (2020). Influence of Nonlocality on Transmittance and Reflectance of Hyperbolic Metamaterials. Crystals.

[45] Kogelnik H., Shank C.V. (1972). Coupled-Wave Theory of Distributed Feedback Lasers. J. Appl. Phys..

[46] Paszkiewicz R., Szczepanski P. (2005). Effect of Excess-Quantum Noise in a One-Dimensional Photonic Crystal Laser. Opt. Commun..

[47] Slooff L.H., van Blaaderen A., Polman A., Hebbink G.A., Klink S.I., Van Veggel F.C.J.M., Reinhoudt D.N., Hofstraat J.W. (2002). Rare-Earth Doped Polymers for Planar Optical Amplifiers. J. Appl. Phys..

